# Neutrophil Inhibitory Factor Selectively Inhibits the Endothelium-Driven Transmigration of Eosinophils *In Vitro* and Airway Eosinophilia in OVA-Induced Allergic Lung Inflammation

**DOI:** 10.1155/2012/245909

**Published:** 2012-12-04

**Authors:** Silvia Schnyder-Candrian, Isabelle Maillet, Marc Le Bert, Lea Brault, Muazzam Jacobs, Bernhard Ryffel, Bruno Schnyder, René Moser

**Affiliations:** ^1^UMR7355, CNRS, Orleans, France; ^2^Experimental and Molecular Immunology and Neurogenetics, University of Orleans, Orleans, France; ^3^Institute for Clinical and Biomedical Research Thurgau, 9548 Matzingen, Switzerland; ^4^Institute of Infectious Disease and Molecular Medicine (IIDMM), University of Cape Town, Cape Town, South Africa; ^5^UAS, HES-SO, Route de Rawyl 47, 1950 Sion, Switzerland; ^6^IBR Inc., Institute for Biopharmaceutical Research, Lauchefeld, 9548 Matzingen, Switzerland

## Abstract

Leukocyte adhesion molecules are involved in cell recruitment in an allergic airway response and therefore provide a target for pharmaceutical intervention. Neutrophil inhibitory factor (NIF), derived from canine hookworm (*Ancylostoma caninum*), binds selectively and competes with the A-domain of CD11b for binding to ICAM-1. The effect of recombinant NIF was investigated. Intranasal administration of rNIF reduced pulmonary eosinophilic infiltration, goblet cell hyperplasia, and Th_2_ cytokine production in OVA-sensitized mice. *In vitro*, transendothelial migration of human blood eosinophils across IL-4-activated umbilical vein endothelial cell (HUVEC) monolayers was inhibited by rNIF (IC_50_: 4.6 ± 2.6 nM; mean ± SEM), but not across TNF or IL-1-activated HUVEC monolayers. Treatment of eosinophils with rNIF together with mAb 60.1 directed against CD11b or mAb 107 directed against the metal ion-dependent adhesion site (MIDAS) of the CD11b A-domain resulted in no further inhibition of transendothelial migration suggesting shared functional epitopes. In contrast, rNIF increased the inhibitory effect of blocking mAbs against CD18, CD11a, and VLA-4. Together, we show that rNIF, a selective antagonist of the A-domain of CD11b, has a prominent inhibitory effect on eosinophil transendothelial migration *in vitro*, which is congruent to the *in vivo* inhibition of OVA-induced allergic lung inflammation.

## 1. Introduction

Allergic asthma has increased dramatically in prevalence and severity over the last two decades. It is a manifold syndrome consisting of bronchospasm and airway hyperreactivity driven by chronic airway inflammation whose underlying immunological mechanisms are still matter of investigation [1, 2]. The accumulation of antigen presenting cells (APCS), Th_2_ lymphocytes, and eosinophils is a prominent feature of chronic airway inflammation [2]. The immigration of leukocytes in the extravascular space is orchestrated by the endothelial barrier and follows an inducible process of stepwise interacting adhesion molecules and chemokines. Intensive research of the last two decades has delineated the molecular program of leukocyte emigration [3–6]. In allergic airway inflammation, the Th_2_ cytokines IL-4 and IL-13 stimulate the endothelium to express VCAM-1 and, by virtue of its binding to very late antigen-4 (VLA-4) [7, 8], facilitate transendothelial migration of eosinophils [9, 10]. The fact that IL-4 and IL-13 do not provoke the endothelial lining to express the neutrophil recruiting E-selectin [11] further explains why eosinophils are preferentially accumulated while neutrophils are often virtually absent in allergic inflammation [12]. As a major counterreceptor of ICAM-1, the *β*_2_-integrin CD11b/CD18 has been associated to neutrophil and macrophage recruitment, although in severe asthma *β*_2_-integrin-expressing lymphocytes and eosinophils are prominent participants [8]. Furthermore, *β*_2_-integrins allow egress of newly generated plasma cells from lymph nodes to the bone marrow [13] while their *in vivo* role in eosinophil recruitment is not thoroughly investigated.

Neutrophil inhibitory factor (NIF) is a 41-kilodalton glycoprotein from the canine hookworm (*Ancylostoma caninum*) that binds to the A-(I)-domain of CD11b/CD18 (*α*M*β*_2_, Mac-1) [14, 15]. Although NIF, a heavily glycosylated polypeptide of 274 amino acids, neither contains the Arg Gly-Asp sequence nor the disintegrin motif, it binds with high specificity and affinity to the A-domain of CD11b/CD18 [15, 16]. The A-domain, is *≈*200 amino acid peptide within the CD11b molecule binding divalent cations and representing a major recognition site for iC3b, fibrinogen, and factor X of the coagulation cascade [17]. By virtue of these multiple but distinct binding sites, the A-domain plays an essential role in phagocytosis, cytotoxicity, and leukocyte trafficking to inflammatory sites.

The function of NIF was so far investigated in *in vivo *models characterized by a dominant neutrophil response. In an acid-induced lung injury model in rabbits, NIF and anti-CD18 antibodies showed comparable attenuation with high number of accumulated neutrophils in the air space. The study indicates that rNIF rather blocks neutrophil cytotoxicity than interfering with their recruitment [18]. In the reverse passive Arthus reaction in rats, neutrophil recruitment depends on CD11a/CD18 and CD11b/CD18 and either of these integrins is sufficient for neutrophil trafficking [19], suggesting that CD11b/CD18 is dispensable for neutrophil recruitment. Given that CD11b/CD18 is expressed on different leukocytes including eosinophils, NIF may also block functions of these leukocytes. With regard to allergic inflammation, eosinophils were in the focus of our interest due to their destructive potential and their preferential accumulation in tissue.

This study shows that rNIF blocks transmigration of eosinophils but not neutrophils across endothelial cell monolayers in culture and inhibits eosinophil recruitment and infiltration, goblet cell hyperplasia, mucus secretion, and Th_2_ cytokine production in the OVA-induced lung inflammation model in mice.

## 2. Material and Methods

### 2.1. Cytokines, Monoclonal Antibodies, and Reagents

Human recombinant IL-1 was kindly provided by Dr. P. T. Lomedica (Hoffmann-La Roche, Nutley, NJ), TNF by Dr. Z. Nagy, (Preclinical Research, Sandoz Ltd., Basel, Switzerland), IL-4 by Dr. J. Banchereau (Schering Plough, Dardilly, France), and recombinant NIF by Dr. Matthew Moyle (Corvas, San Diego, CA, USA). The ELISA kits for mouse IL-4, IL-5 were from R&D (Abingdon, UK). The mAbs IB-4 (IgG2a) recognizing CD18, mAb 60.1 (IgG1) against CD11b and H12 (IgG1) directed to CD11a were generous gifts of Dr. S. Wright (Rockefeller University, New York, USA). The mAb CLB54 against CD18 was kindly provided by Dr. J. Ghrayeb, Centocor Inc., Malvern, PA, USA. The mAbs HP2/1 (IgG1) against VLA-4 (CDw49d) and 25.3 (IgG1) against CD11a were from Immunotech S. A., Marseille, France. The mAb W6/32 (anti-HLA frames structure; Sera-Lab, Crawley Down, England) was used as negative control. The mAb 107 was a generous gift of Dr. M. A. Arnaout (Massachusetts General Hospital, Charlestown, MA 02129, USA). In functional inhibition experiments, all mAbs were used at proven saturating concentrations of 10 *μ*g/mL as described [20].

O-phenylenediamine, 3-amino-1,2,4-triazole, horseradish peroxidase, and chicken egg albumin grade V were obtained from Sigma Chemical Company (St. Louis, MO).

### 2.2. Mice and Experimental Protocol

BALB/c mice were bred in our specific pathogen-free animal facility at CNRS, Orleans, France. Mice were maintained in a temperature-controlled (23°C) facility with a strict 12-hour light/dark cycle and were given free access to food and water. The experiments were performed with gender-matched mice aged 6 to 8 weeks. All protocols complied with the French Government's ethical and animal experiment regulations.

Mice were immunized subcutaneously twice at weekly interval with 0.2 mL saline containing 10 *μ*g ovalbumin (OVA) with 1.6 mg aluminium hydroxide. One week after the second immunization, mice were challenged 3 times (at days 14, 15, and 16) as follows. Animals were hold under light i.v. ketamine, xylazine anesthesia and administered intranasally with 40 *μ*L saline (0.9%) containing 10 *μ*g OVA alone, or OVA with 25 *μ*g rNIF. Control mice were challenged with saline alone, or 25 *μ*g rNIF alone, given in 40 *μ*L saline solution. One day after the last challenge, plethysmography analysis was performed. Two days after the last challenge mice were sacrificed and the lungs analyzed.

Mice were given a high dose of ketamine/xylazine i.p. and bled out. Via a tracheal cannula, the lungs were washed twice with 1 mL of ice-cold saline (see below Bronchoalveolar lavage fluid). After bronchoalveolar lavage, the lung was perfused via heart puncture with ISOTON II acid-free balanced electrolyte solution (Beckman Coulter, Krefeld, Germany). Lungs were fixed overnight in buffered 4% formaldehyde solution for histology analysis. BAL fluid was analyzed for cell composition and cytokine concentrations. Experiments were performed at least twice using groups of 4 animals.

### 2.3. Airway Resistance Using Whole-Body Plethysmography

Bronchial hyperreactivity (BHR) to aerosolized methacholine was investigated at 24 h after the last OVA challenge. Unrestrained conscious mice were placed in whole-body plethysmography chambers (Buxco Electronic, Sharon, CO, USA). Mice were exposed for 50 seconds to 100 mM methacholine. The constriction was measured for 15 min after nebulization. Mean airway bronchoconstriction was estimated by the enhanced respiratory pause (Penh) index. Penh can be conceptualized as the phase shift of the thoracic flow and the nasal flow curves; increased phase shift correlates with increased respiratory system resistance. Penh is calculated by the formula Penh = (Te/RT − 1) × PEF/PIF, where Te is expiratory time, RT is relaxation time, PEF is peak expiratory flow, and PIF is peak inspiratory flow. For the graphics, the Penh mean values are given for −3 min to −1 min (baseline) and 14 points or minutes after the methacholine nebulization.

### 2.4. Bronchoalveolar Lavage (BAL)

Bronchoalveolar lavages (BAL) fluids were prepared by washing the lungs 4 times with 0.5 mL of ice-cold saline. The cells were sedimented by centrifugation at 400 ×g for 10 min at 4°C. The supernatants (cell-free BAL fluid) were stored at −70°C for cytokine analysis. An aliquot of the cell pellets was stained with Trypan Blue solution, counted, and 200,000 cells centrifuged on microscopic slides (cytospin at 1000 rpm for 10 min, at RT). Air-dried preparations were fixed and stained with Diff-Quik (Merz & Dade AG, Dudingen, Switzerland). Differential counts were made under oil immersion microscopy. Two times one hundred cells were counted for the determination of the relative percentage of each cell type present in the BAL.

### 2.5. Lung Histology

The organs were fixed in 4% buffered formaldehyde overnight and embedded in paraffin as described previously [21]. Lung sections of 3 *μ*m were stained with hematoxylin and eosin or with periodic acid Schiff reagent (PAS) and examined with a Leica microscope (×40 and ×100 magnification). Peribronchial eosinophil infiltration and goblet cell hyperplasia with mucus hypersecretion were assessed by a semiquantitative score (0–5) by two observers independently.

### 2.6. Quantitation of IL-4 and IL-5 in BAL

Cytokine concentrations in BAL were determined by enzyme-linked immunosorbent assay (ELISA), using commercial kits from R&D (Abingdon, UK). The cytokine detection limit was 1 pg/mL.

### 2.7. Recombinant NIF

Recombinant NIF was expressed in Chinese hamster ovary (CHO) cells and purified from conditioned cell supernatant by immunoaffinity chromatography as described [15]. Stock solutions of purified recombinant NIF (herein called as rNIF) were in PBS, pH 7.3 at an approximate concentration of 10 mg/mL.

### 2.8. Endothelial Cell Cultures

HUVECs were harvested as previously described [22]. The cells were seeded on purified human fibronectin (Winiger AG, Wohlen, Switzerland) and grown in medium 199 enriched with sodium heparin (90 *μ*g/mL; Novo Industries, Copenhagen, Denmark) and endothelial cell growth supplement (15 *μ*g/mL; Collaborative Research, Inc., St. Waltham, MA) in the presence of 20% pooled human serum. Final monolayers were used in their second to fourth passage exhibiting the cytoplasmic factor VIII von Willebrand as tested by indirect immunofluorescence with rabbit anti-human factor VIII Ab [23].

### 2.9. Preparation of Bilayer Vascular Constructs

Bilayer vascular constructs consisting of confluent HUVEC layers on the top basal layers of extracellular matrix from human fibroblasts were prepared as described [9]. Briefly, human lung fibroblasts were cultured in MEM alpha containing 10% of FCS for 10 d in 24-well plates. The resulting multilayers were washed once with PBS and lysed with 1 mL/well of a 0.5% aqueous solution of ammonium. The remaining extracellular matrix layers were rinsed 5 times with PBS, before the HUVECs were seeded in a 1 : 2 ratio and grown in complete culture medium until they reached confluence.

### 2.10. Purification of Neutrophils and Eosinophils

Granulocytes were separated from 400 mL of heparinized (20 U/mL) blood; (Novo Industries, Copenhagen, Denmark) whole blood from normal individuals by methocel-metrizoate sedimentation and subsequent buoyant density centrifugation over Ficoll-Paque (Pharmacia, Uppsala, Sweden) as previously described [22]. The resulting sediment was, essentially free from monocytes and lymphocytes and contained mostly neutrophils (96–99%) and eosinophils (1–4%). Eosinophils were further enriched by negative selection using CD 16 immunomagnetic beads according to the method of Hansel et al. [24] and were cultured overnight in Iscove's medium containing 10% FCS, 10 pM recombinant human GM-CSF, and 10 pM recombinant IL-3 (Sandoz Ltd., Basel, Switzerland) as described [20]. The activated eosinophils accumulated between distinct layers of a discontinuous Percoll gradient at densities between 1.070 g/mL and 1.075 g/mL. This behavior characterizes the metabolically activated hypodense phenotype found in allergic diseases [20].

### 2.11. Determination of Eosinophil and Neutrophil Transendothelial Migration

Bilayer vascular constructs were preincubated with cytokines at indicated concentrations for 4 h or 18 h at 37°C, washed twice with HBSS, and coincubated for 2 h at 37°C together with 2 × 10^5^ neutrophils or eosinophils resuspended in 500 *μ*L of MEM-*α* medium containing 10% FCS with or without NIF and/or mAbs at indicated concentrations. Transendothelial migration was determined after 2 hours as described [9, 10]. In brief, the bilayers were fixed with 1% paraformaldehyde in PBS-A and transmigrated eosinophils were counted in a blind fashion in 4 arbitrarily chosen high power fields focused on a subendothelial plane. The number of eosinophils/high power field (0.25 mm^2^) was calculated as the percentage of migrated eosinophils.

### 2.12. Statistical Analysis

Statistical validation was performed using Student's two-tailed *t*-test for unpaired observations. *P*  values of less than 0.05 are considered statistically different.

## 3. Results

### 3.1. Recombinant NIF Decreases Eosinophil Recruitment and Th_2_ Cytokines in OVA-Challenged BALB/c Mice

The heterodimeric *β*_2_-integrin CD11b/CD18 is expressed on most leukocytes including eosinophils participating in the airway hyperresponsiveness of allergic asthma. Disruption of the *β*_2_-integrin-ICAM-1 interaction by rNIF may attenuate the allergic airway inflammation. Therefore, the potential inhibitory effect of rNIF together with the antigen challenge in immunized mice was investigated and the allergic response analyzed.

**Figure 1:**
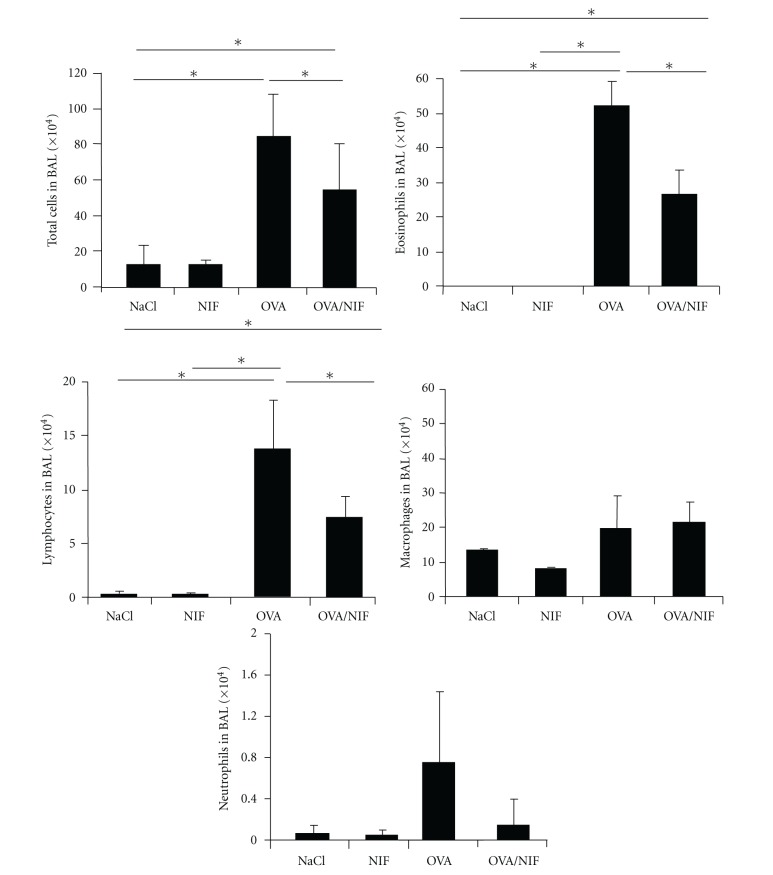
Recombinant NIF inhibits lung eosinophil infiltration, and Th_2_ cytokine release. Immunized BALB/c mice were challenged intranasaly with either saline (NaCl), rNIF alone (NIF), OVA alone (OVA), or the combination OVA with 25 *μ*g rNIF (OVA/NIF). Forty-eight hours after the third challenge, bronchoalveolar lavage (BAL) was performed and the cell composition determined. The total numbers of eosinophils (dotted bars), lymphocytes (black bars), macrophages (hatched bars), and neutrophils (white bars) are presented. Figure represents means ± SD of 6 animals per group (* indicates *P* < 0.05).

In the absence of rNIF total BAL (bronchoalveolar lavage) cell counts increased up to 24-fold in OVA-challenged BALB/c mice as compared to saline treatment (15 × 10^4^ to 354 × 10^4^ cells/BAL). The composition of the OVA-induced leukocytes in the BAL (Figure 1) was dominated by invading eosinophils (*P* < 0.01) and lymphocytes (*P* < 0.01). In contrast, macrophages were not significantly increased compared to the saline control, and the number of recruited neutrophils in OVA-challenged BALB/c mice was negligible (Figure 1). Recombinant NIF when given by the intranasal route at 25 *μ*g per mouse with the OVA challenge reduced the total BAL cell counts by 40% (*P* < 0.01), with a 50% reduction of eosinophils (*P* < 0.01) and lymphocytes. Macrophages numbers in the BAL were not reduced by rNIF (Figure 1). 

**Figure 2:**
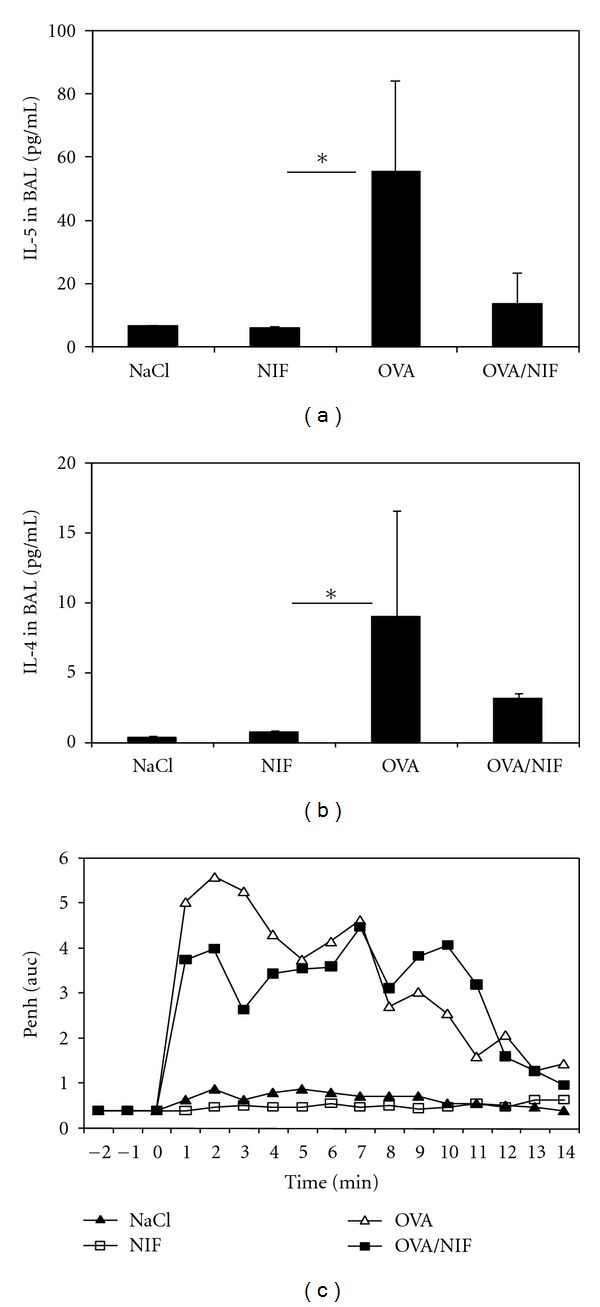
The presence of rNIF reduced IL-5 and IL-4 secretion into the BAL but not acute bronchial hyperreactivity. Forty-eight hours after the third challenge, bronchoalveolar lavage (BAL) was performed. The concentrations of IL-5 (*P* < 0.05; (a)) and IL-4 (ns; (b)) were measured by ELISA. Bronchial hyperreactivity (BHR) to methacholine nebulization was determined 24 h before sacrificing using whole-body plethysmography (c). BHR intensity was measured in Penh arbitrary units after indicated challenges and expressed as AUC values. The results represent means ± SD (*n* = 8 animals per group; * indicates *P* < 0.05).

In addition, production of IL-4 and IL-5 was determined in the BAL samples. Ova challenge induced a significant increase of IL-4 and IL-5 in BAL fluid, while IL-13 could not be detected. Intranasal administration of rNIF reduced OVA-induced IL-5 and IL-4 production in the BAL fluid by 80% and 75%, respectively (Figures 2(a) and 2(b)). 

To investigate the effect on airways hyperresponsiveness rNIF was given by the intranasal route at a dose of 25 *μ*g together with each antigen challenge in OVA-sensitized BALB/c mice. OVA-challenge of OVA immunized BALB/c mice, but not NaCl or rNIF alone, developed a robust response to aerosolized methacholine expressed as enhanced respiratory pause (Penh) values as assessed by whole-body plethysmography (Figure 2(c)). Penh values provide an estimate for airway obstruction and may indicate airway hyperreactivity. The CD11b antagonist NIF when given together with the OVA challenge revealed no significant inhibition of the methacholine response (Figure 2(c)) as calculated by AUC assessment of Penh values over time.

### 3.2. Recombinant NIF Decreases Mucus Hypersecretion and Eosinophil Recruitment in the Lung Tissue

**Figure 3:**
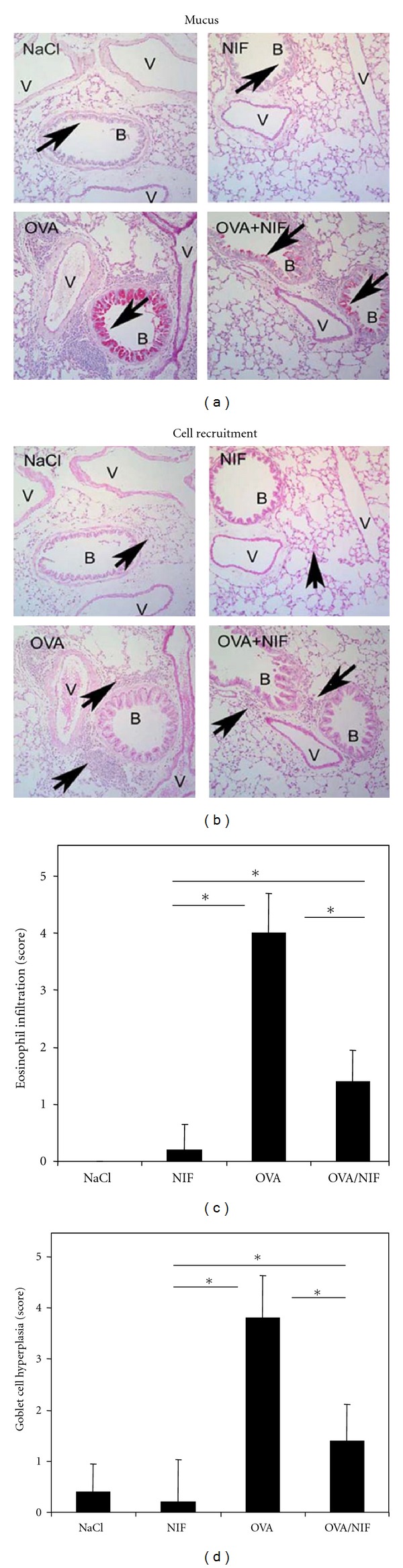
Role of rNIF in cell recruitment to the lungs and mucus hypersecretion. Lung sections of BALB/c mice sacrificed two days after the third challenge with either saline (NaCl), rNIF alone (NIF), OVA alone (OVA), and OVA with rNIF (OVA/NIF) are shown. The formalin-fixed tissue sections were stained with periodic acid Schiff reagent (PAS) to visualize mucus (a) and with hematoxylin/eosin to visualize cell recruitment (b), as shown by the arrows. Representative lung sections are shown at magnification ×20. V: blood vessel; B: bronchioles. Eosinophil recruitment (c) and goblet cell hyperplasia with mucus hypersecretion (d) were assessed semiquantitatively. Results represent means ± SD (*n* = 5 animals per group; * indicates *P* < 0.05).

The morphological hallmarks of allergic asthma are peribronchial eosinophilic inflammation, mucus overproduction, and bronchial smooth muscle cell hyperplasia. OVA challenge in immunized mice caused distinct peribronchial cell recruitment with eosinophils together with hyperplasia of bronchial smooth muscle and increased mucus production. Recombinant NIF alone had no effect (data not shown), but drastically reduced OVA-induced mucus hypersecretion and hyperplasia of goblet cells and peribronchial eosinophil infiltration (Figures 3(a) and 3(b)). The effect of rNIF on OVA-induced eosinophil infiltration and mucus overproduction (goblet cell hyperplasia) was assessed semiquantitatively. Upon OVA challenge rNIF significantly reduced both eosinophil recruitment (Figure 3(c)) and mucus hypersecretion (*P* < 0.05), while rNIF on its own had no effect (Figure 3(d)). Together, the data suggest that the CD11b antagonist NIF inhibits OVA-induced allergic inflammation *in vivo*.

### 3.3. Recombinant NIF Inhibits Transendothelial Migration of Eosinophils

As rNIF significantly reduces eosinophil infiltration in the peribronchial space and the cytokine-activated endothelium is known to govern leukocyte trafficking in inflammation, it was of interest whether rNIF would inhibit the passage of eosinophils across endothelial layers *in vitro*.

In these experiments, human blood eosinophils were conditioned with GM-CSF and IL-3 for 24 h to induce changes mimicking the active phenotype found in allergic inflammation [20].

**Figure 4:**
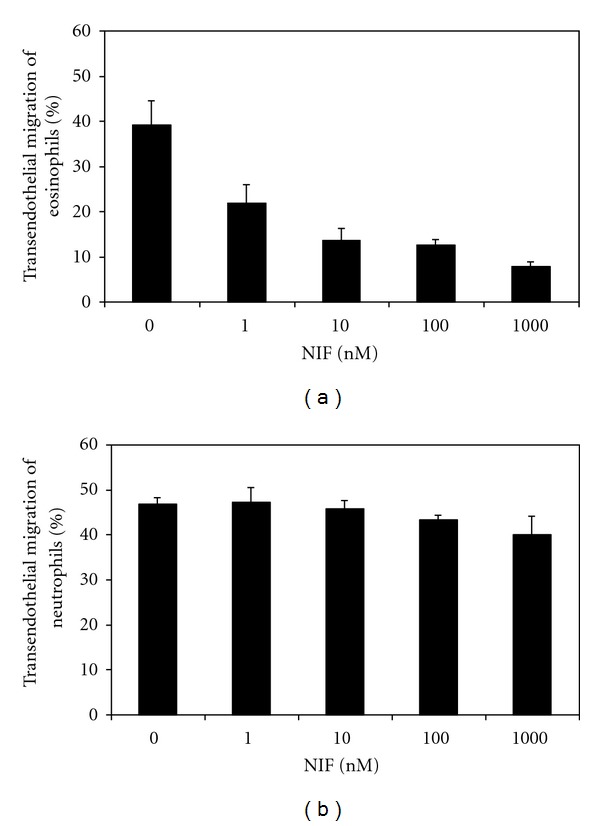
Recombinant NIF inhibits transendothelial migration of eosinophils but not neutrophils. Bilayer vascular constructs were activated with 1 ng/mL IL-4 for 16 h before transendothelial migration of eosinophils was determined in the presence of indicated concentrations of rNIF (a). In separate experiments bilayer vascular constructs were preincubated with 10 ng/mL TNF for 4 h. Thereafter, transendothelial migration of neutrophils was determined in the presence of indicated concentrations of rNIF (b). Graphs depict means of triplicate determinations ± SD representing typical results of a series of 3 independent experiments.

Bilayer vascular constructs were used to determine transendothelial migration (TEM) *in vitro*. They consisted of HUVEC monolayers grown on extracellular matrix from human fibroblast multilayers [9]. Pretreatment of the bilayers with IL-1 and TNF preferentially provoked TEM of neutrophils [9]. Conversely, IL-4-pretreated bilayers selectively induced TEM of eosinophils [9]. Considering these cell type specific conditions for transmigration, we investigated the impact of rNIF on TEM of eosinophils and neutrophils in a series of separate experiments (Figure 4). TEM of eosinophils and neutrophils across unstimulated bilayer vascular constructs was 3.1 ± 0.6% and 3.2 ± 0.5%, respectively (means of triplicate determinations ±SD of a representative experiment). With IL-4-pretreated bilayers a more than 10-fold increase of the TEM of eosinophils was observed which was inhibited in a dose-dependent manner by rNIF (Figure 4(a)). Half maximal inhibition of TEM (IC_50_) was obtained at 4.6 ± 2.6 nM rNIF (mean ± SEM of three experiments). In contrast, TEM of neutrophils provoked by TNF stimulation of the bilayers was not significantly impaired by rNIF even at concentrations up to 1 *μ*M (Figure 4(b)).

**Figure 5:**
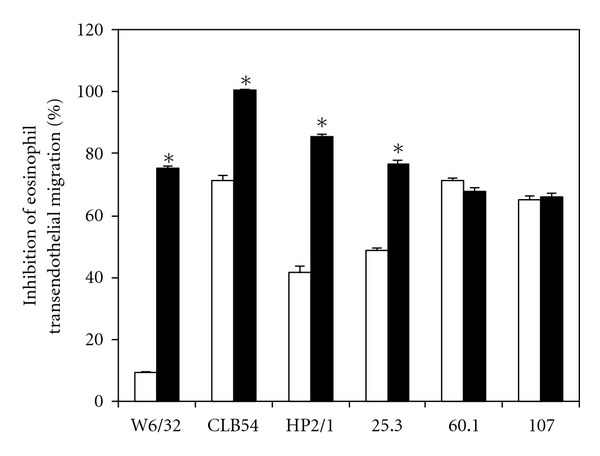
Effects of blocking mAbs in the presence of rNIF on transendothelial migration of eosinophils across IL-4activated HUVEC monolayers. Eosinophils were either incubated with the control mAb W6/32 (anti HLA *α*-chain) or CLB54 (anti-CD18), HP2/1 (anti-VLA-4), 25.1 (anti-CD11a), 60.1 (anti-CD11b), or 107 (anti-CD11b MIDAS) alone (white bars) or with 10 nM rNIF (black bars). All mAbs were used at saturating concentrations of 10 *μ*g/mL. Results are means of triplicate determinations ± SD of a representative example out of a series of three experiments (* indicates *P* < 0.05).

Although the process of eosinophil emigration depends on CD11b/CD18, our data indicate that the interaction is more complex involving different adhesion molecules. Therefore, the inhibiting property of rNIF was further investigated together with blocking mAbs against CD18 (CLB54), VLA-4 (HP2/1), and CD11a (25.3; Figure 5). In the presence of saturating concentrations of these mAbs TEM of eosinophils was partially inhibited. The inhibitory effect conferred by the mAbs CLB54, HP2/1, and 25.3 was considerably enhanced by rNIF indicating independent blocking mechanisms. In contrast, the finding that rNIF showed no additional inhibition in the presence of mAb 60.1 suggests shared functional epitopes (Table 1). Similar data to using mAb 60.1 were obtained, when mAb 107 against the metal ion-dependent adhesion site (MIDAS) of the CD11b A-domain was applied.

In summary, the experiments allow the conclusion that rNIF inhibits TEM of eosinophils by binding to the MIDAS region in the A-domain of CD11b. These data are congruent to our *in vivo* observation that rNIF significantly reduces accumulation of eosinophils in the peribronchial space.

## 4. Discussion

Blood eosinophilia is closely associated to parasitosis representing the first line of defense. Once attached to targets, such as larvae of schistosomula mansoni, eosinophils kill by producing large amounts of cationic proteins and oxidative metabolites [25]. Blood eosinophilia is also a hallmark of allergic inflammation, and similar mechanisms may drive eosinophil recruitment and accumulation in the tissue [26]. The discovery and characterization of a potent neutrophil inhibiting factor, a 41 kD glycoprotein from the canine hookworm (*Ancylostoma caninum*) [14], sheds light to a parasitic survival strategy and provides a concept to attenuate allergic inflammatory response of the host. Whereas many studies have characterized the potential of rNIF to antagonize neutrophil activation and thereby treat postischemic inflammation [27–30], there are no studies published that are focusing on allergic inflammation.

Here we show that rNIF has a dominant antiallergic potential. In OVA-immunized BALB/c mice, rNIF given by the intranasal route with the OVA challenge strongly reduced the number of eosinophils and lymphocytes in the BAL. Furthermore, in the presence of rNIF, IL-4 and IL-5 secretion in the airways was reduced, which may be linked to the diminished recruitment of eosinophils and lymphocytes. Recombinant NIF also reduced mucus secretion and hyperplasia of goblet cells in OVA-induced lung inflammation. In contrast, antigen-specific IgE production was not impaired (data not shown) suggesting that T and B cell interactions established during OVA immunization are not disturbed by rNIF. Similarly, rNIF had no influence on the airway hyperresponsiveness when given together with the OVA challenge.

At the endothelial barrier the process of transmigration orchestrates composition and localization of the leukocyte infiltrate. We and others have shown that IL-4 and IL-13 activate endothelial cells to express VCAM-1 and provoke transmigration of eosinophils and other VLA-4-expressing leukocytes [9, 10, 31, 32]. Recombinant NIF inhibited the IL-4-provoked transendothelial migration of eosinophils in a dose-dependent fashion.

CD18, VLA-4, and CD11a are crucially involved in the transmigration process [4]. Blocking mAbs against these ligands partially blocked eosinophil transmigration, and rNIF enhanced the inhibitory effects providing independent blocking mechanisms. The fact that rNIF binds specifically and with high affinity to recombinant constructs containing to the A-domain of CD11b [15, 16] explains these observation. In contrast, rNIF together with mAb 60.1 against CD11b showed no extra inhibition. Similar data were obtained when rNIF was incubated together with mAb 107 against the metal ion-dependent adhesion site (MIDAS) of the CD11b A-domain. The mAb 60.1 is known to specifically inhibit the adherence of activated neutrophils to nonstimulated endothelium while not impairing adhesion of nonactivated neutrophils to cytokine activated endothelium [33, 34]. The mAb 107 has been shown to preferentially bind to the inactive low-affinity form of the CD11b A-domain, and it was suggested that its antagonistic effect is exerted in part by stabilizing the receptor in the low-affinity state [35]. Together, these data indicate that distinct epitopes in the A-domain are functional in the interaction leading to transmigration of eosinophils. Surprisingly, even at concentrations up to 1 *μ*M rNIF did not inhibit TEM of neutrophils across TNF or IL-1-activated endothelial cells. These experiments cast doubt on the potency of rNIF to block neutrophil invasion in acute inflammation. 

Still, the A-domain of CD11b is crucial for neutrophil spreading, chemotaxis, adhesion-dependent degranulation, and superoxide generation and rNIF substantially inhibits these functions. In addition, adherence of activated neutrophils and eosinophils to unstimulated endothelial cells in culture is CD11b/CD18 dependent [36–39]. All these interactions mediated by activated granulocytes are strongly inhibited by rNIF [14]. On the other hand, non-activated neutrophils interact with cytokine-activated endothelial cells in a sequential interaction leading to transendothelial migration *in vitro* and to the rapid localization of neutrophils in microbial infections and acute inflammation [5]. Although the A-domain of CD11b is indispensable (for review see [40]), its function is complex involving both inside-out and outside-in signals that convert conformational changes in integrins to control ligand binding affinity [41]. Given that rNIF binds specifically and with high affinity to the A-domain of CD11b our data support the evidence that the blocking function of NIF is restricted to distinct functional epitopes and does not imply a total blockade of the A-domain [15, 16].

Together, the study shows congruent *in vivo* and *in vitro* data suggesting that rNIF operates at the crossroad of antiparasitic defense and allergic inflammation by interfering with the recruitment of eosinophils at the endothelial barrier and thereby modulates allergic inflammation.

## Figures and Tables

**Table 1:** Cumulative effects of blocking mAbs in the presence of rNIF on transendothelial migration of eosinophils across IL-4-activated HUVEC monolayers.

Antigen		Inhibition of eosinophil transendothelial migration (%)			
	mAb	Buffer		rNIF 10 nM	
		Mean	SD	Mean	SD
HLA I *α* chain	W6/32	9.1	0.7	75.2	0.9
CD18	CLB54	71.3	1.9	100.4	0.4
VLA-4	HP2/1	41.7	2.2	85.7	0.9
CD11a/CD18	25.3	48.7	1.0	76.5	1.2
CD11b/CD18	60.1	71.3	0.9	67.8	1.2
CD11b A-domain MIDAS	107	65.2	1.0	66.1	1.0

Eosinophils were either incubated with the indicated mAbs alone or together with 10 nM rNIF. Data are given as mean of triplicate determinations ±SD.

## Abbreviations

BAL: Bronchoalveolar lavage
EPO: Eosinophil peroxidase
rNIF: Recombinant Neutrophil Inhibitory Factor
TEM: Transendothelial migration.
